# Hematopoietic or Osteoclast-Specific Deletion of Syk Leads to Increased Bone Mass in Experimental Mice

**DOI:** 10.3389/fimmu.2019.00937

**Published:** 2019-04-30

**Authors:** Dániel Csete, Edina Simon, Ahmad Alatshan, Petra Aradi, Csaba Dobó-Nagy, Zoltán Jakus, Szilvia Benkő, Dávid S. Győri, Attila Mócsai

**Affiliations:** ^1^Department of Physiology, Semmelweis University School of Medicine, Budapest, Hungary; ^2^Department of Physiology, Faculty of Medicine, University of Debrecen, Debrecen, Hungary; ^3^MTA-SE “Lendület” Lymphatic Physiology Research Group of the Hungarian Academy of Sciences and the Semmelweis University, Budapest, Hungary; ^4^Department of Oral Diagnostics, Semmelweis University School of Dentistry, Budapest, Hungary

**Keywords:** SYK (spleen tyrosine kinase), tyrosine kinase, osteoclasts, Cre-Lox, *in vivo*, mice

## Abstract

Syk is a non-receptor tyrosine kinase critically involved in signaling by various immunoreceptors including B-cell-receptors and activating Fc-receptors. We have previously shown that Syk also mediates immunoreceptor-like signals required for the *in vitro* development and function of osteoclasts. However, the perinatal lethality of *Syk*^−/−^ mice precluded the analysis of the role of Syk in *in vivo* bone metabolism. To overcome that problem, we generated mice with osteoclast-specific (*Syk*^Δ*OC*^) or hematopoietic (*Syk*^Δ*Haemo*^) Syk deficiency by conditional deletion of Syk using Cre recombinase expressed under the control of the Ctsk or Vav1 promoter, respectively. Micro-CT analysis revealed increased bone trabecular density in both *Syk*^Δ*OC*^ and *Syk*^Δ*Haemo*^ mice, although hematopoietic Syk deficiency caused a more severe phenotype than osteoclast-specific Syk deficiency. Osteoclast-specific Syk deficiency reduced, whereas hematopoietic Syk deficiency completely blocked *in vitro* development of osteoclasts. Both interventions inhibited the resorptive activity of osteoclasts and osteoclast-specific gene expression. Kinetic analysis of Syk protein levels, Cre expression and the genomic deletion of the *Syk*^flox^ allele revealed complete and early deletion of Syk from *Syk*^Δ*Haemo*^ osteoclasts whereas Syk was incompletely deleted at a later stage of osteoclast development from *Syk*^Δ*OC*^ cultures. Those results provide an explanation for the *in vivo* and *in vitro* difference between the *Syk*^Δ*OC*^ and *Syk*^Δ*Haemo*^ mutant strains and suggest late activation of, and incomplete target gene deletion upon, osteoclast-specific Cre expression driven by the Ctsk promoter. Taken together, our results indicate that Syk plays an indispensable role in osteoclast-mediated *in vivo* bone resorption and suggest that Syk-specific inhibitors may provide therapeutic benefit in inflammatory and other diseases characterized by excessive osteoclast-mediated bone resorption.

## Introduction

Osteoclasts are multinuclear giant cells of hematopoietic origin which develop from myeloid progenitors through a unique biochemical maturation program followed by homotypic fusion (1, 2). Osteoclasts are the sole cell types in the mammalian organism capable of actively resorbing bone tissue and therefore play a critical role in bone homeostasis. Defective osteoclast development or function leads to increased bone mass (osteopetrosis) (3), whereas excessive (pathological) bone resorption occurs during osteoporosis (4), inflammatory joint diseases (e.g., arthritis-induced bone erosions in rheumatoid arthritis) (5, 6) and cancer-induced bone loss (7, 8).

Osteoclast development and function requires a number of extracellular cues including M-CSF, RANKL, as well as integrin-mediated adhesive processes (9). The importance of those pathways is indicated by the severe bone resorption defects in mice lacking M-CSF (10), RANK (11, 12), RANKL (13, 14), or β_3_ integrins (15). Culturing myeloid progenitors derived from human blood or mouse bone marrow in the presence of M-CSF and RANKL also leads to formation of osteoclast-like cells with *in vitro* bone resorbing capacity, allowing the analysis of osteoclast development and function in cell culture.

Syk is a non-receptor tyrosine kinase critically involved in various functions of the immune system, as well as certain non-immune-related biological processes (16). Syk is required for B-cell-receptor signaling and therefore the development of B-cells (17, 18). It is a critical component of signaling by a number of activating Fc-receptors such as Fcε-receptors and Fcγ-receptors on neutrophils, macrophages, and mast cells (19–22), as well as the Fc-receptor-related collagen receptor GpVI of platelets (23, 24). Syk also mediates signaling by β_1_, β_2_, and β_3_ integrins in neutrophils, monocytes/macrophages, and platelets (25–27). Syk deficiency causes perinatal lethality (17, 18) likely due to the role of Syk in lymphatic vascular development (28). Most, if not all of those functions of Syk is related to its binding to receptor-associated tyrosine-phosphorylated immunoreceptor tyrosine-based activation motifs (ITAMs) linking immunoreceptors to downstream signaling pathways (16, 29–32). The role of Syk in various immune and inflammatory processes also translates into its role in autoantibody-induced arthritis (24, 33–35) and dermatitis (36, 37) in experimental mice.

We and others have previously shown that the ITAM-containing adapter molecules DAP12 and FcRγ are involved in *in vitro* osteoclast development and function, and that mice lacking both DAP12 and FcRγ show strongly increased mineralized bone mass (38–43). One of the possible mechanisms for those phenotypes could be that, similar to immune cells (16, 29), the ITAM-containing DAP12 and FcRγ adapters would activate the Syk tyrosine kinase in osteoclasts, thus triggering osteoclast development and function. Indeed, Syk-deficient bone marrow cells failed to develop to mature multinucleated osteoclasts or to show resorptive activity in *in vitro* cultures (40, 42, 44, 45), and this *in vitro* phenotype was linked to ITAM signaling by DAP12 and FcRγ (42–44). Those studies provided an unexpected link between immunoreceptor-like signaling and bone homeostasis and therefore provided one of the foundations of the field of osteoimmunology (46, 47). In addition, Syk-mediated pathways have also been linked to integrin signal transduction and the osteoclast cytoskeleton (16, 26, 42, 44, 48). Unfortunately, however, it is at present unclear whether Syk is also involved in bone homeostasis in live animals, as bone morphology of Syk-deficient animals could not be tested because of the perinatal lethality of *Syk*^−/−^ mice (17, 18).

To overcome the perinatal lethality of *Syk*^−/−^ animals, we have generated mice with osteoclast-specific or hematopoietic-specific Syk deletion using the Cre-Lox recombination approach. Analysis of the mice with tissue specific Syk deletion revealed strong increase in bone mass upon osteoclast-specific and, particularly, hematopoietic-specific Syk-deficiency, indicating a critical role for Syk in *in vivo* bone homeostasis. Further experiments aimed at understanding the different severities of the bone phenotypes in the two strains indicated that the effect of Syk deficiency on osteoclast development strongly depends on the timing and extent of Cre expression and Cre-mediated inactivation of the *Syk* gene.

## Materials and Methods

### Animals

Mice carrying the *Syk*^tm1.2Tara^ (referred to as *Syk*^flox^) floxed allele of the *Syk* gene (49) were obtained from Alexander Tarakhovsky (Rockefeller University) and were maintained in homozygous (*Syk*^flox/flox^) form. Mice carrying the *Ctsk*^tm1(cre)Ska^ (referred to as *Ctsk*^Cre^) knock-in mutation resulting in the osteoclast-specific expression of the Cre recombinase under the control of the endogenous promoter of the *Ctsk* gene and at the same time inactivating the *Ctsk* gene (50) were obtained from Shigeaki Kato (University of Tokyo) and were maintained in heterozygous form (referred to as Ctsk-Cre) to avoid homozygous inactivation of the *Ctsk* gene. Mice carrying the *Commd10*^Tg(Vav1−icre)A2Kio^ transgenic insertional mutation expressing the Cre recombinase in the entire haemopoietic lineage from the exogenous Vav1 promoter (51) and at the same time inactivating the *Commd10* gene (52) were obtained from the Jackson Laboratory and were maintained in heterozygous form (referred to as Vav-Cre) to avoid homozygous inactivation of the *Commd10* gene. Mice carrying the *Lyz2*^tm1(cre)Ifo^ (referred to as *Lyz2*^Cre^) knock-in mutation expressing the Cre recombinase in the entire myeloid compartment from the endogenous promoter of lysozyme M (53) were purchased from the Jackson Laboratory and were maintained in homozygous form (referred to as LysM-Cre).

Osteoclast-specific deletion of *Syk* was achieved by crossing the Ctsk-Cre and *Syk*^flox/flox^ mice to obtain *Ctsk*^Cre/+^*Syk*^flox/flox^ (referred to as *Syk*^Δ*OC*^) animals. Deletion of Syk in the entire hematopoietic compartment was achieved by crossing the Vav-Cre and *Syk*^flox/flox^ mice to obtain *Commd10*^Tg(Vav1−icre)A2Kio/+^*Syk*^flox/flox^ (referred to as *Syk*^Δ*Haemo*^) animals. Myeloid-specific deletion of Syk was achieved by crossing the LysM-Cre and *Syk*^flox/flox^ mice to obtain *Lyz2*^*Cre*^^/Cre^*Syk*^flox/flox^ (referred to as *Syk*^Δ*Myelo*^) animals. The allele obtained by Cre-mediated deletion of the *Syk*^flox^ allele will be referred to as the *Syk*^Δ^ allele.

Genotyping of the mice was performed by allele-specific PCR. All mice were on the C57BL/6 genetic background. Wild type C57BL/6 animals were obtained from our breeding colony. The mice were kept in individually sterile ventilated cages (Tecniplast) in a specific pathogen-free facility. All animal experiments were approved by the Animal Experimentation Review Board of Semmelweis University.

### Micro-CT Analysis

Mice were sacrificed at 9 weeks of age and their right femurs were subjected to micro-CT analysis by a SkyScan 1172 micro-CT apparatus as described (54, 55). A 70 kV and 124 μA X-ray source with 0.5 mm aluminum filter and a rotation step of 0.5° was used during image acquisition, followed by reconstruction with the SkyScan NRecon software, resulting in an isometric 5 μm voxel size. Volume of interest was selected according to the manufacturer's instructions. Further analysis was performed using the Skyscan CTAn and CTVol software. The lower threshold of binary images was set to an absolute value of 85 throughout the entire study. Our study design did not allow the calculation of absolute bone hydroxyapatite densities.

Quantitative analysis was performed on the trabecular region of the distal femoral metaphysis beginning 50 sections (0.25 mm) from the distal growth plate to an additional 400 sections (2 mm) to the proximal direction, including the entire trabecular area within that range, identified manually by visual inspection. Quantitative parameters included percent bone volume (BV/TV), trabecular number, trabecular thickness and trabecular separation as described (54, 55).

Representative cross sections represent the 200th section (1 mm) from the distal femoral growth plate. 3D images show an axial cylinder of a diameter of 500 μm between sections 150–450 from the distal growth plate.

### Histological Procedures and Immunostaining

Femurs isolated from mice at 9 weeks of age were fixed in 4% paraformaldehyde (Sigma-Aldrich) followed by decalcification in Osteomoll (Merck) for 3 weeks. The samples were then dehydrated, and embedded in paraffin (Leica) using a Leica EG1150H embedding station. Eight micrometers of thick sections were obtained using a Thermo Scientific HM340E microtome and were processed for hematoxylin and eosin (Leica) staining, or for immunostaining for the calcitonin receptor using anti-Calcitonin Receptor (Abcam AB11042) and anti-rabbit Alexa Fluor 488 (Life Technologies, A11034) antibodies. Microscopic images were taken by a Nikon ECLIPSE Ni-U microscope connected to a Nikon DS-Ri2 camera.

### *In vitro* Culture and Resorption Assays

*In vitro* osteoclast cultures were performed essentially as described before (54, 55). Bone marrow cells obtained by flushing the tibia and femur of wild type or mutant mice were cultured in the presence of 10 ng/ml murine M-CSF (Peprotech) for 2 days in α-MEM medium (Sigma) supplemented with 10% FCS (Gibco) and antibiotics. Non-adherent cells were then plated at the concentration of 1.5 × 10^5^ cells/cm^2^ and cultured in the presence of 50 ng/ml recombinant murine M-CSF and 50 ng/ml murine RANKL (Peprotech) with medium changes every 2 days. In parallel macrophage cultures, the cells were cultured under identical conditions except that RANKL was omitted.

Cultures were terminated and osteoclast-specific staining was performed using a commercial tartrate-resistant acid phosphatase (TRAP) staining kit (Sigma-Aldrich) at the indicated times after the first addition of RANKL. Photomicrographs were taken using a Leica DMI6000B inverted microscope. The images were then analyzed either manually or by the ImageJ software. Osteoclasts were defined as TRAP-positive cells with 3 or more nuclei.

For *in vitro* resorption assays, osteoclasts were cultured under similar conditions for 7 days on an artificial hydroxyapatite surface (Sigma-Aldrich) followed by washing, imaging by dark field microscopy and further analysis by ImageJ software.

### Biochemical Studies

For protein content analysis, osteoclast, or macrophage cultures were washed and then lysed in a Triton-based lysis buffer containing 100 mM NaCl, 30 mM Na-HEPES (pH 7.4), 20 mM NaF, 1 mM Na-EGTA, 1% Triton X-100, 1 mM benzamidine, freshly supplemented with 0.1 U/ml Aprotinin, 1:100 Mammalian Protease Inhibitor Cocktail, 1:100 Phosphatase Inhibitor Cocktail 2, 1 mM PMSF, and 1 mM Na_3_VO_4_ (all from Sigma-Aldrich). Insoluble material was removed, the lysate supernatants were supplemented with 4× Laemmli's sample buffer and boiled for 10 min. Whole cell lysates were run on SDS-PAGE, electroblotted to nitrocellulose membranes, and then processed for immunoblotting with antibodies against Syk (N19; Santa Cruz) or β-actin (Clone AC-74; Sigma-Aldrich). After incubation with peroxidase-labeled secondary antibodies (GE Healthcare), the signal was developed using the ECL system (GE Healthcare) and exposed to X-ray film. X-ray films were then scanned and processed with Adobe Photoshop.

### Quantitative RT-PCR Analysis

To test osteoclast specific and Cre gene expression changes, mouse myeloid progenitors were differentiated into osteoclasts or macrophages in the presence of 50 ng/ml M-CSF with or without 50 ng/ml RANKL for 0–3 days, followed by RNA extraction and reverse transcription as previously described (54–56). For quantitative reverse transcription (RT)-PCR analysis of the osteoclast-specific genes, the following TaqMan assays were used: *Acp5* (TRAP; Taqman Mm00475698_m1), *Ctsk* (cathepsin K; Mm00484039_m1), *Calcr* (Calcitonin receptor; Mm00432271_m1), *Nfatc1* (NFATc1; Mm00479445_m1), and *Tm7sf4* (DC-STAMP; Mm04209235_m1) as previously described (54, 55). For assessment of Cre expression, the 5′- TGACGGTGGGAGAATGTTAATC forward and 5′ GCTACACCAGAGACGGAAATC reverse primers were used. Transcript levels relative to GAPDH were calculated using the comparative Ct method (54, 55).

### Sequencing of the Germline *Syk*^flox^ Allele

To determine the exact sequence of the *Syk*^flox^ allele, tail DNA was amplified using the 5′- GCC CGT TCT GTG CCT ACT GG−3′ forward and 5′- TAG CTA ACC AAA CCC ACG GC−3′ reverse primers spanning the 5′ loxP site, or the 5′- CCA AAG CGG AGT CCT CAC AT−3′ forward and 5′- GTC GGT CCC ATC TTT CC−3′ reverse primers spanning the 3′ loxP site. PCR products were then sent to Microsynth for sequencing and the obtained sequences were aligned with the genomic sequence of the wild type *Syk* gene to obtain the sequence of the *Syk*^flox^ allele.

### Genomic PCR Analysis

Osteoclast cultures were washed at the indicated times after the start of RANKL treatment, followed by isolation of genomic DNA and PCR using standard procedures.

Two different PCR assays were performed on the genomic DNA of osteoclast cultures. In PCR 1, the 5′- GCC CGT TCT GTG CCT ACT GG−3′ forward primer (P fwd) was used along with the 5′- TAG CTA ACC AAA CCC ACG GC−3′ reverse primer (P rev1) to separate the *Syk*^+^ and Syk^flox^ alleles (234 and 349 bp product length, respectively). In PCR 2, the same P fwd forward primer was used with the 5′- GTC GGT CCC ATC TTT CC−3′ reverse primer (P rev2) to separate the *Syk*^+^, *Syk*^flox^ and *Syk*^Δ^ alleles (1314, 1560 and 452 bp product length, respectively).

### Statistical Analysis

Experiments were performed the indicated number of times. Diagrams show mean and SEM from the indicated number of independent experiments. Micro-CT measurements were analyzed by two-way (factorial) ANOVA with the presence/absence of Cre and the *Syk* genotype as the independent parameters. Other measurements were analyzed by one-way ANOVA followed by Tukey or Unequal n HSD *post hoc* test. In case of the kinetic analysis of osteoclast morphology, statistical analysis was performed on the area under the curve (AUC). *P*-values below 0.05 were considered statistically significant.

## Results

### The Effect of Osteoclast-Specific Syk Deletion on Trabecular Bone Architecture

The *Syk*^−/−^ mutation causes perinatal lethality making it technically impossible to analyze the bone morphology of adult *Syk*^−/−^ mice. We decided to overcome that problem by generating lineage-specific Syk-deficient animals. As a first approach, we crossed mice in which the cDNA of the Cre recombinase has been inserted into the osteoclast-specific Ctsk gene (referred to as *Ctsk*^Cre/+^ or Ctsk-Cre mice) (50) with mice carrying a floxed *Syk* allele (referred to as *Syk*^flox/flox^ mice) (49). The resulting *Ctsk*^Cre/+^*Syk*^flox/flox^ (referred to as *Syk*^Δ*OC*^) mice are expected to have defective Syk expression in osteoclasts due to Cre-mediated excision and inactivation of the Syk gene.

**Figure 1:**
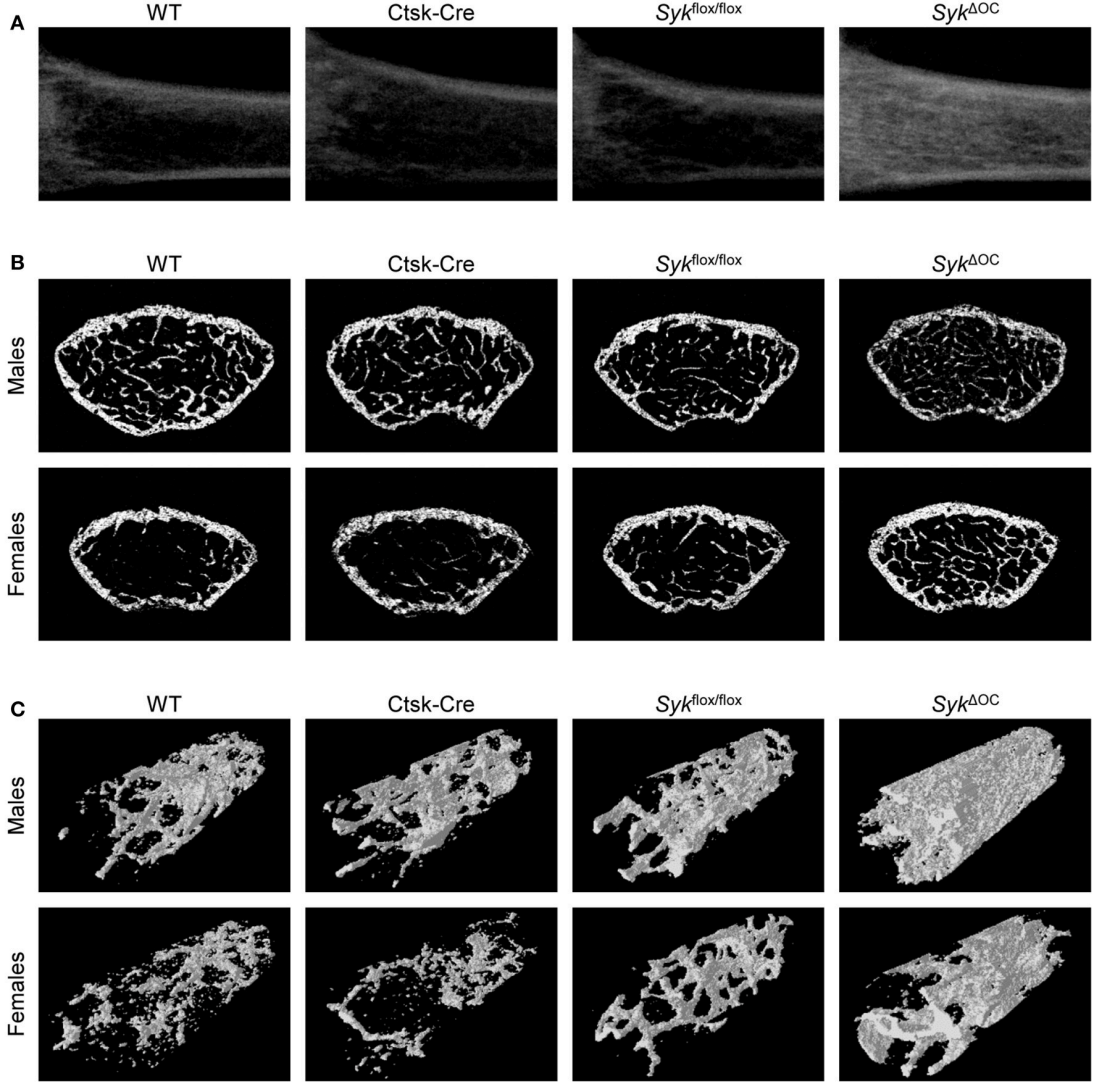
Micro-CT analysis of osteoclast-specific Syk-deficient mice. Representative micro-CT images of the femurs of 9-week-old mice of wild type (WT) and the indicated mutant mice. **(A)** Longitudinal sections of the femur of female mice. **(B)** Cross-sections of the femur of male or female mice. **(C)** 3D reconstitution of an axial cylinder of the trabecular area of the distal metaphysis of femurs of male or female mice. Images are representative of micro-CT analysis of 5 mice per gender and genotype.

We then subjected *Syk*^Δ*OC*^ mice and the appropriate controls to micro-CT analysis of the distal femur. As shown in the longitudinal sections of the femurs of female mice in Figure 1A, the *Syk*^Δ*OC*^ mutation strongly increased the density of the trabecular area compared to wild type mice, whereas no dramatic difference could be observed in Ctsk-Cre or *Syk*^flox/flox^ animals. Analysis of representative cross-sections of male or female mouse femurs also showed increased trabecular density in *Syk*^Δ*OC*^ but not in Ctsk-Cre or *Syk*^flox/flox^ animals, particularly in the case of female mice (Figure 1B). The increased trabecular density was also evident in three-dimensional reconstitution of an axial cylinder within the trabecular area of the femurs (Figure 1C).

**Figure 2:**
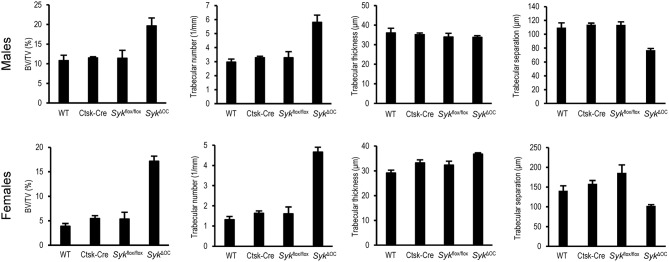
Quantitative micro-CT analysis of osteoclast-specific Syk deficiency. The right femurs of 9-week-old wild type (WT) or the indicated mutant male or female mice were subjected to micro-CT analysis, followed by quantification of percent bone volume (bone volume/total volume, BV/TV), trabecular number, trabecular thickness and trabecular separation. The graphs show mean and SEM of data obtained from 5 mice per gender and genotype.

We also processed micro-CT images for quantitative analysis, incorporating data from the entire trabecular space within a defined distance range from the distal femoral growth plate. As shown in Figure 2, the percent bone volume (BV/TV) was strongly increased in *Syk*^Δ*OC*^ mice, whereas no substantial difference could be observed in Ctsk-Cre or *Syk*^flox/flox^ mice. Male wild type mice had an ~2.8-fold higher (10.8%) basal percent bone volume (BV/TV) than their female counterparts (3.9%). However, the increase in BV/TV in *Syk*^Δ*OC*^ over wild type mice was more robust in female (4.4-fold) than in male (1.8-fold) animals (Figure 2). We have also performed statistical analysis by two-way (factorial) ANOVA which determines the interaction of the two (Ctsk-Cre and *Syk*^flox/flox^) mutations, i.e., whether the co-existence of the two mutation in the *Syk*^Δ*OC*^ resulted in a statistically significant difference beyond an additive effect. That analysis revealed a significant increase of the BV/TV values both in male (*p* = 0.028) and, especially, in female (*p* = 0.00005) mice.

Further quantitative (Figure 2) and statistical (two-way ANOVA) analysis of the trabecular bone revealed a higher trabecular number in *Syk*^Δ*OC*^ mice (*p* = 0.0069 and 0.00001 for males and females, respectively), whereas no consistent change was observed in the trabecular thickness of the same animals (*p* = 0.85 and 0.87 for males and females, respectively). In agreement with the increased trabecular number, trabecular separation was reduced in *Syk*^Δ*OC*^ mice (*p* = 0.00032 and 0.0011 for males and females, respectively).

Taken together, our results indicate that osteoclast-specific deletion of Syk causes increased bone trabecular mass primarily due to increased bone trabecular number rather than a higher trabecular thickness. However, the phenotype observed in *Syk*^Δ*OC*^ mice (Figure 2) appeared to be less dramatic than that reported for *Tyrobp*^−/−^*Fcer1g*^−/−^ double knockout mice lacking both the DAP12 and FcRγ ITAM-containing adapter molecules which were previously proposed to signal through Syk (42, 43, 57).

### The Effect of Hematopoietic Deletion of Syk on Trabecular Bone Architecture

The apparently less severe bone phenotype of *Syk*^Δ*OC*^ mice compared to *Tyrobp*^−/−^*Fcer1g*^−/−^ (DAP12/FcRγ double knockout) animals (42, 43, 57) could either be due to a less critical role for Syk in *in vivo* bone homeostasis or the less complete deletion of Syk in *Syk*^Δ*OC*^ animals. To test this latter possibility, we turned to mice with Syk deficiency in the entire hematopoietic compartment due to deletion by the Vav-Cre transgene which causes Cre expression during the early stages of hematopoiesis (51). Accordingly, we subjected Vav-Cre *Syk*^flox/flox^ (referred to as *Syk*^Δ*Haemo*^) mice and appropriate controls to microCT analysis of the distal femur.

**Figure 3:**
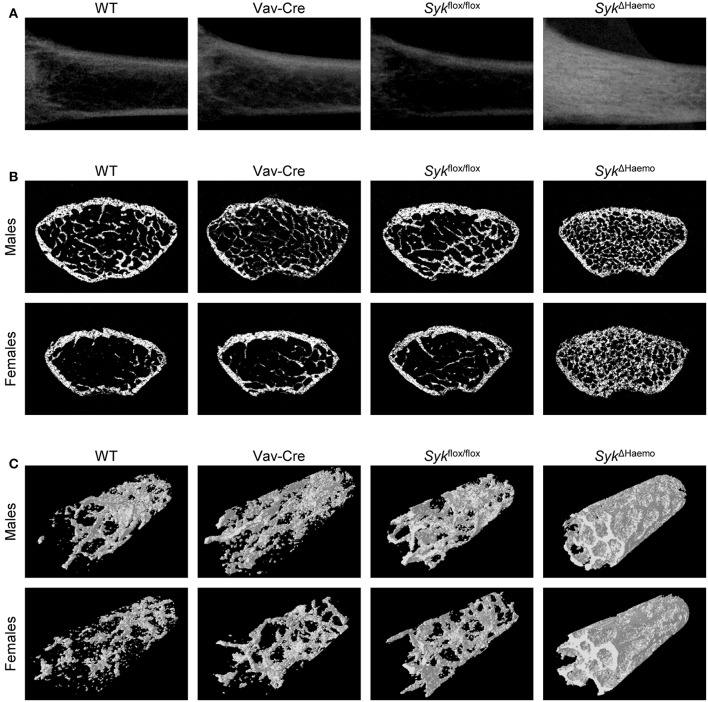
Micro-CT analysis of hematopoietic Syk-deficient mice. Representative micro-CT images of the femurs of 9-week-old mice of wild type (WT) and the indicated mutant mice. **(A)** Longitudinal sections of the femur of female mice. **(B)** Cross-sections of the femur of male or female mice. **(C)** 3D reconstitution of an axial cylinder of the trabecular area of the distal metaphysis of femurs of male or female mice. Images are representative of 5–7 mice per gender and genotype. WT samples are identical to those shown in Figure 1.

As shown in Figure 3A, Syk deletion in the entire hematopoietic compartment by the *Syk*^Δ*Haemo*^ mutation caused a very strong increase in trabecular density in the longitudinal sections of the femurs of female animals, whereas no substantial changes were observed in Vav-Cre or *Syk*^flox/flox^ mice. An increased trabecular density in *Syk*^Δ*Haemo*^ mutants could also be observed in cross-sections of the distal femurs of male and, in particular, female mice, whereas no obvious differences could be seen in Vav-Cre or *Syk*^flox/flox^ animals (Figure 3B). Three-dimensional reconstitution of a trabecular area cylinder also showed visible increases in the trabecular density in *Syk*^Δ*Haemo*^ animals (Figure 3C).

**Figure 4:**
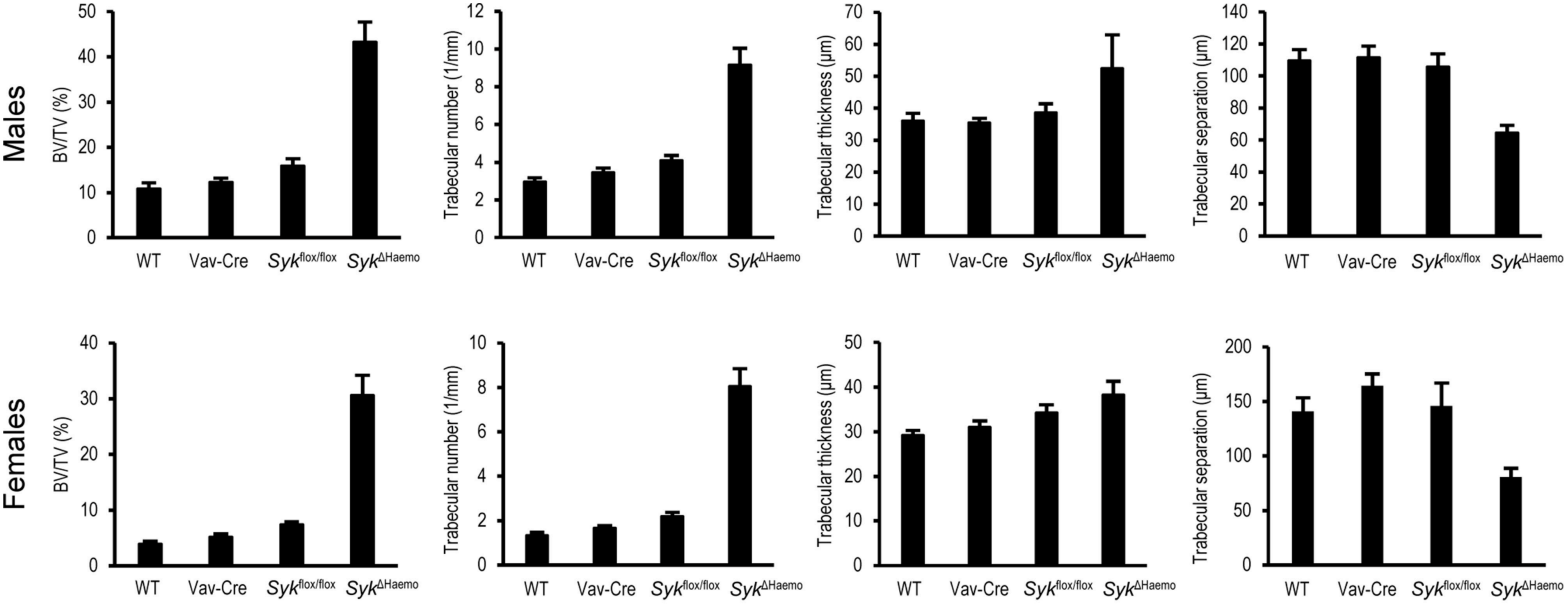
Quantitative micro-CT analysis of hematopoietic Syk deficiency. The right femurs of 9-week-old wild type (WT) or the indicated mutant male or female mice were subjected to micro-CT analysis, followed by quantification of percent bone volume (bone volume/total volume, BV/TV), trabecular number, trabecular thickness and trabecular separation. The graphs show mean and SEM of data obtained from 5–7 mice per gender and genotype. WT values are identical to those shown in Figure 2.

Further quantitative analysis of the microCT data (Figure 4) indicated a strongly increased percent bone volume (BV/TV) in *Syk*^Δ*Haemo*^ mice in both male and female animals. Importantly, BV/TV values in *Syk*^Δ*Haemo*^ mice appeared to be substantially higher than corresponding *Syk*^Δ*OC*^ animals (compare Figures 2, 4). On the other hand, similar to the *Syk*^Δ*OC*^ results, the BV/TV fold increase in *Syk*^Δ*Haemo*^ over wild type animals was higher in females (7.9-fold) than in males (4.0-fold), again primarily due to the higher basal values in male wild type mice. Statistical analysis by two-way ANOVA revealed a highly significant interaction between the effects of the Vav-Cre and *Syk*^flox/flox^ mutations (*p* = 0.00032 and 0.00003 for males and females, respectively), indicating that Cre-mediated deletion of Syk in *Syk*^Δ*Haemo*^ mice strongly increases trabecular bone mass.

Further quantitative assessment (Figure 4) and statistical analysis (two-way ANOVA) revealed that, similar to the *Syk*^Δ*OC*^ mice, the increased trabecular bone volume was primarily due to an increased trabecular number (*p* = 0.0010 and 0.00001 for males and females, respectively), rather than significant changes in trabecular thickness (*p* = 0.31 and 0.61 for males and females, respectively). Trabecular separation was also reduced in *Syk*^Δ*Haemo*^ mice (*p* = 0.0045 and 0.0071 for males and females, respectively).

Taken together, early deletion of Syk in the entire hematopoietic system results in dramatic increase in the mineralized trabecular bone mass, indicating a critical role for Syk in *in vivo* bone homeostasis. The bone phenotype seen in *Syk*^Δ*Haemo*^ mice is grossly comparable to that reported for *Tyrobp*^−/−^*Fcer1g*^−/−^ (DAP12/FcRγ double knockout) animals (42, 43, 57), raising the possibility that the majority of DAP12/FcRγ signals proceeds through Syk in live mice. However, the 30–45% BV/TV values observed in *Syk*^Δ*Haemo*^ mice are substantially higher than the corresponding values (15–20%) in *Syk*^Δ*OC*^ animals, raising the possibility that the lower values in the latter mutants may be due to incomplete deletion of Syk by Cre expression from the Ctsk-Cre mutation.

### Bone Histological Analysis

**Figure 5:**
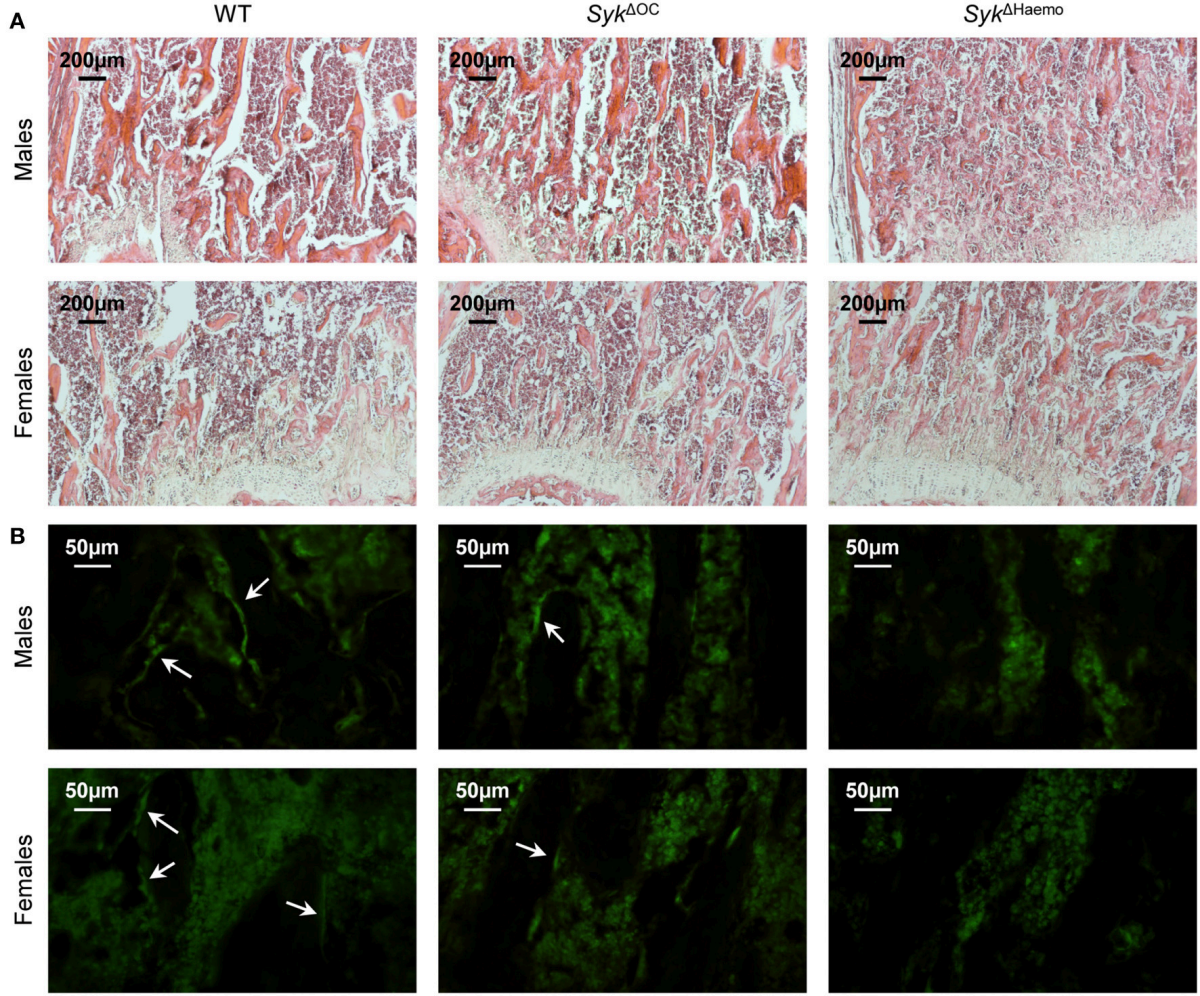
Histological and immunofluorescence analysis of osteoclast-specific and hematopoietic Syk deficiency. Representative photomicrographs of the trabecular area of the femurs of 9-week-old wild type (WT), *Syk*^Δ*OC*^ or *Syk*^Δ*Haemo*^ mice. **(A)** Haematoxylin and eosin staining; original magnification ×10. **(B)** Calcitonin receptor immunostaining; original magnification ×40. Arrows indicate calcitonin receptor-positive bone lining cells (likely osteoclasts). Images are representative of 3 mice per gender and genotype.

We have also performed histological analysis of the distal femur of wild type, *Syk*^Δ*OC*^ or *Syk*^Δ*Haemo*^ mice. As shown in Figure 5A, a much more dense trabecular network was seen in *Syk*^Δ*OC*^ and, especially, *Syk*^Δ*Haemo*^ mice than in wild type animals. Again, the difference was more pronounced in female mice because of the lower trabecular density in female than in male mice in the wild type cohorts.

To test the presence of mature osteoclasts on the trabecular bone surface, we have performed immunofluorescence staining of bone sections for calcitonin receptor, an osteoclast-specific differentiation marker. As shown in Figure 5B, calcitonin receptor signals were evident on the lining of trabecular rods (dark areas) in wild type sections. Similar signals were also seen but at substantially lower numbers in *Syk*^Δ*OC*^ sections, whereas no such signals were seen in *Syk*^Δ*Haemo*^ sections (Figure 5B). Those results suggest that the number of calcitonin receptor-positive osteoclasts is reduced in *Syk*^Δ*OC*^ and, especially, in *Syk*^Δ*Haemo*^ mice.

### *In vitro* Osteoclast Development in Lineage-Specific Syk Mutants

We next tested *in vitro* development of osteoclasts from wild type, *Syk*^Δ*OC*^ or *Syk*^Δ*Haemo*^ bone marrow cells in the presence of recombinant M-CSF and RANKL cytokines. Bone marrow cells were first cultured for 2 days in low (10 ng/ml) M-CSF and non-adherent cells (referred to as myeloid progenitors) were then cultured in the presence of 50 ng/ml M-CSF and 50 ng/ml RANKL. Osteoclast development was then tested by assessing cell morphology and positive histochemical staining for the osteoclast-specific TRAP enzyme.

**Figure 6:**
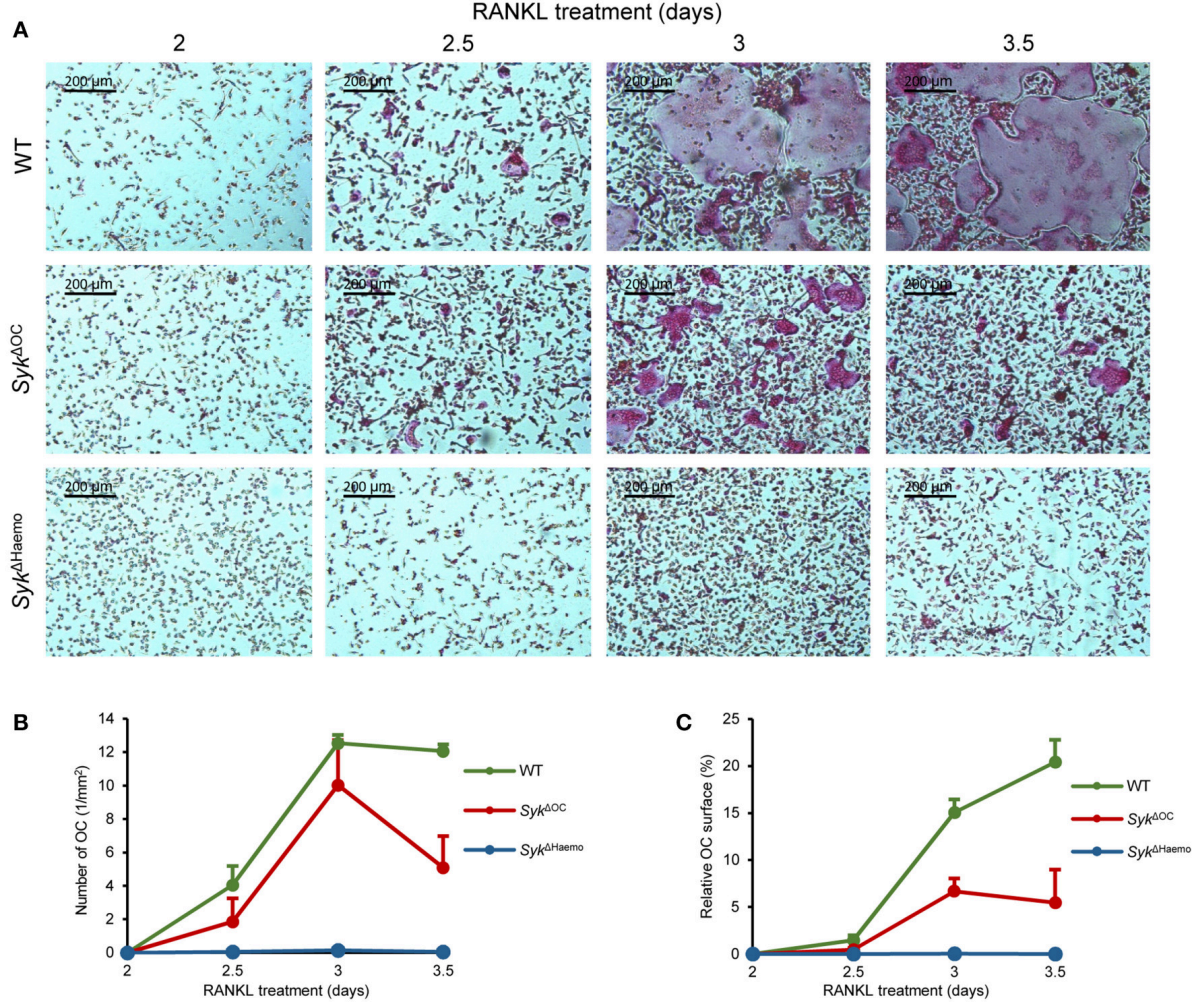
Analysis of *in vitro* osteoclast development. Bone marrow-derived myeloid progenitors from wild type (WT), *Syk*^Δ*OC*^ or *Syk*^Δ*Haemo*^ mice were cultured in the presence of 50 ng/ml M-CSF and 50 ng/ml RANKL for the indicated times, followed by staining for tartrate-resistant acid phosphatase (TRAP). **(A)** Representative images of TRAP-stained cultures. **(B)** Quantification of the number of osteoclasts (TRAP-positive cells with 3 or more nuclei) in the different cultures. **(C)** The area covered by osteoclasts in the different cultures (in % of the total culture area). Images are representative of, and bar graphs show mean and SEM from, 3 independent experiments.

As shown in Figure 6A, no TRAP-positive multinuclear cells (osteoclasts) were seen 2 days after addition of RANKL to the cultures. However, osteoclasts started to appear in wild type cultures on day 3 and formed very large multinucleated TRAP-positive cells 3.5 days after the initial RANKL treatment. Some osteoclasts also formed in *Syk*^Δ*OC*^ cultures, though they were much smaller in size and failed to fuse into very large cells even by 3.5 days after RANKL treatment (Figure 6A). On the other hand, practically no osteoclasts (multinucleated TRAP-positive cells) could be observed in *Syk*^Δ*Haemo*^ cultures (Figure 6A).

We have also quantitated the extent of *in vitro* osteoclast formation. To this end, we have counted the number of osteoclasts (defined as TRAP-positive cells with 3 or more nuclei; Figure 6B) and calculated the percent of the culture area covered by the osteoclasts (Figure 6C). Though the two different quantification approaches were related to each other, they also complemented each other, since later stages of osteoclast development may lead to the emergence of very large osteoclasts which occupy large culture areas but are small in numbers (as seen in the last two images in wild type cultures in Figure 6A).

As seen in Figures 6B,C, there were practically no osteoclasts in any of the cultures 2 days after the initial RANKL addition. However, osteoclasts rapidly emerged afterwards in wild type cultures, reaching a maximum number 1 day later. The area covered by wild type osteoclasts increased further in the next 12 h, even though the number of osteoclasts started to decline, indicating the fusion of the cells into a few very large osteoclasts in this final stage of osteoclast development (Figures 6B,C). The number of osteoclasts also increased in *Syk*^Δ*OC*^ cultures and was temporarily even comparable to that of wild type osteoclasts (Figure 6B). However, those *Syk*^Δ*OC*^ osteoclasts covered a significantly smaller area than in wild type cultures throughout the experiments (Figure 6C), which was in line with the smaller size of *Syk*^Δ*OC*^ osteoclasts in Figure 6A. On the other hand, again in agreement with the photomicrographs in Figure 6A, practically no osteoclasts could be identified in *Syk*^Δ*Haemo*^ cultures (Figures 6B,C).

We have also performed more detailed statistical analyses (one-way ANOVA) of the area under the curve (AUC) from data presented in Figures 6B,C. In case of the number of osteoclasts (Figure 6B), no statistical difference was seen between the wild type and *Syk*^Δ*OC*^ cultures (*p* = 0.12), likely reflecting the fact that the osteoclast numbers only declined on the last day in the *Syk*^Δ*OC*^ samples (Figure 6B). However, the number of osteoclasts in the *Syk*^Δ*Haemo*^ cultures was statistically highly significantly reduced compared to wild type ones (*p* = 0.0013). The total area covered by osteoclasts was highly significantly reduced both by the *Syk*^Δ*OC*^ (*p* = 0.00058) and the *Syk*^Δ*Haemo*^ (*p* = 0.00024) mutations.

The above results confirm prior studies indicating a critical role for Syk during *in vitro* osteoclast development (40, 42, 44). On the other hand, they also indicate an incomplete osteoclast developmental defect in *Syk*^Δ*OC*^ cultures (as opposed to the complete defect in *Syk*^Δ*Haemo*^ ones), suggesting incomplete deletion of Syk in *Syk*^Δ*OC*^ mutants.

### Analysis of the *in vitro* Resorptive Activity of Osteoclasts

We also attempted to test the *in vitro* resorbing capacity of osteoclasts. To this end, myeloid precursors were plated on an artificial hydroxyapatite layer and cultured in the presence of M-CSF and RANKL (50 ng/ml each) for 7 days, followed by assessment of hydroxyapatite resorption by dark field microscopy. It should be noted that this assay measures the combined effect of both osteoclast development and osteoclast-mediated matrix resorption.

**Figure 7:**
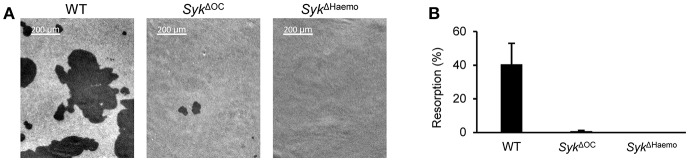
Analysis of the *in vitro* resorptive function of osteoclasts. Bone marrow-derived myeloid progenitors from wild type (WT), *Syk*^Δ*OC*^ or *Syk*^Δ*Haemo*^ mice were cultured in the presence of 50 ng/ml M-CSF and 50 ng/ml RANKL for 7 days on an artificial hydroxyapatite layer. **(A)** Representative dark-field microscopic images of resorption pits (dark areas). **(B)** Quantification of the resorption area (in percent of the total area). Images are representative of, and bar graphs show mean and SEM from, 3 independent experiments.

As shown in Figure 7A, wild type osteoclast cultures were able to resorb substantial areas of the hydroxyapatite layer (resorbed areas show a dark appearance). In contrast, only small areas of resorption could be observed in *Syk*^Δ*OC*^ cultures and no resorption was seen in *Syk*^Δ*Haemo*^ cultures (Figure 7A). Quantification of the resorbed area revealed ~40% resorption in wild type cultures, which was strongly reduced by the *Syk*^Δ*OC*^ and completely eliminated by the *Syk*^Δ*Haemo*^ mutations (Figure 7B). Statistical analysis (one-way ANOVA) revealed highly significant reduction of the resorption activity both by the *Syk*^Δ*OC*^ (*p* = 0.00040) and the *Syk*^Δ*Haemo*^ (*p* = 0.00038) mutations.

These results confirm an important role for Syk in the development and/or function of bone-resorbing osteoclasts (42), and also indicate slight differences between the *Syk*^Δ*OC*^ and *Syk*^Δ*Haemo*^ mutations.

### Analysis of Osteoclast-Specific Gene Expression

**Figure 8:**
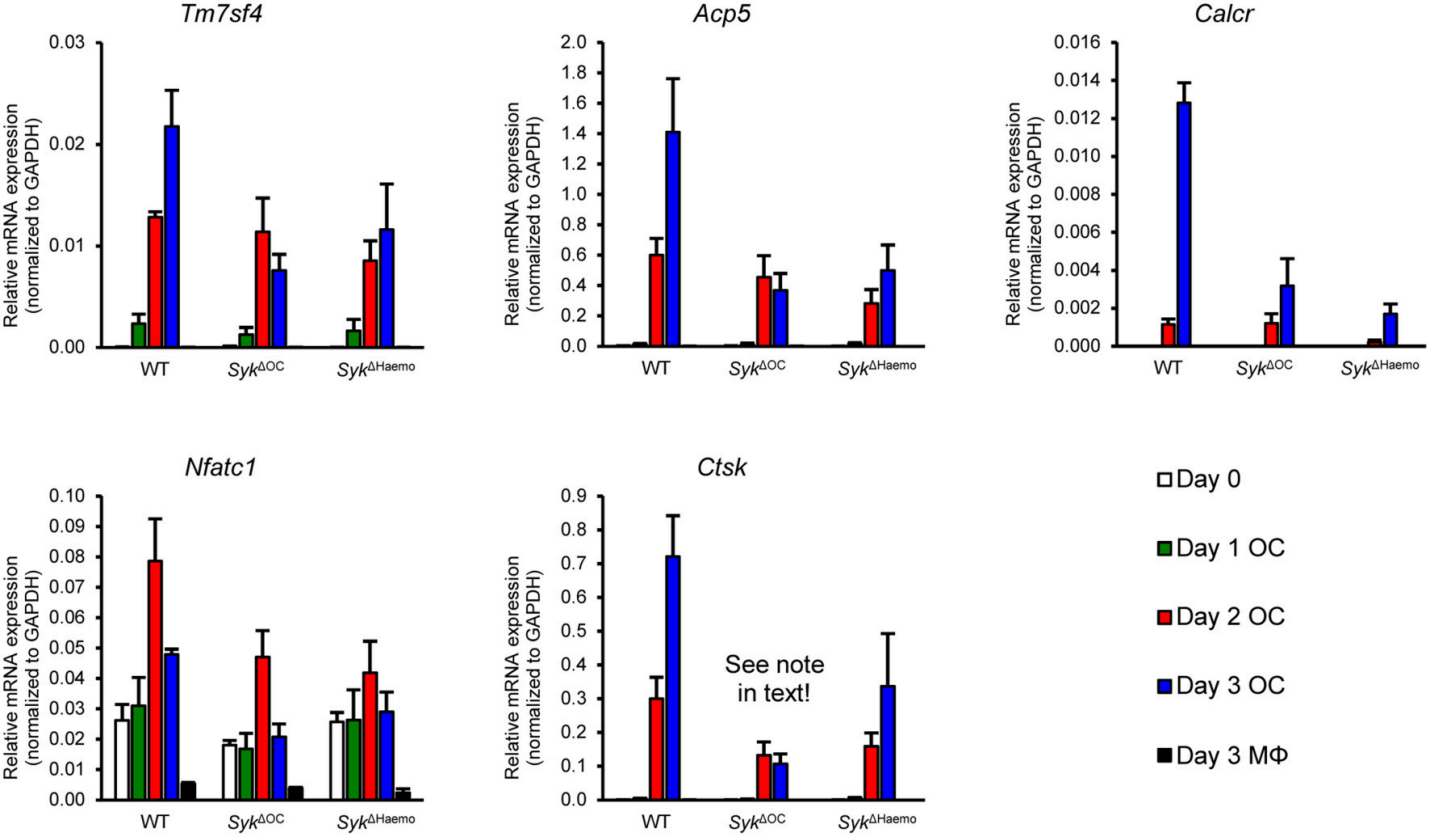
Analysis of osteoclast-specific gene expression. Gene expression in bone marrow-derived cells from wild type (WT), *Syk*^Δ*OC*^ or *Syk*^Δ*Haemo*^ mice cultured for 0–3 days in the presence of 50 ng/ml M-CSF with (osteoclasts; OC) or without (macrophages; MΦ) 50 ng/ml RANKL. The expression of the *Tm7sf4, Acp5, Calcr, Nfatc1*, and *Ctsk* genes (encoding for DC-STAMP, TRAP, Calcitonin receptor, NFATc1, and Cathepsin K, respectively) were determined by quantitative RT-PCR and normalized to *Gapdh*. Bar graphs show mean and SEM from 3 independent experiments.

We next tested the changes of osteoclast-specific gene expression in osteoclast cultures from the different genotypes. We have also tested additional control macrophage cultures generated under identical conditions except that RANKL treatment was omitted. As shown in Figure 8, the expression of DC-STAMP (encoded by the *Tm7sf4* gene), TRAP (*Acp5*), calcitonin receptor (*Calcr*), NFATc1 (*Nfatc1*) and cathepsin K (*Ctsk*) mRNA strongly increased upon osteoclastic differentiation whereas no such increase could be observed in parallel macrophage cultures. The expression of all those genes were reduced in both the *Syk*^Δ*OC*^ and *Syk*^Δ*Haemo*^ cultures (Figure 8), though the defect ranged from a moderate (*Tm7sf4*) to a very strong (*Calcr*) reduction. It should also be noted that the reduced expression of *Ctsk* in *Syk*^Δ*OC*^ samples is likely partially due to the inactivation of one of the two alleles of the *Ctsk* gene by the Ctsk-Cre (*Ctsk*^Cre/+^) mutation present in those cells. Taken together, gene expression data indicate a role for Syk in regulation of osteoclast-specific gene expression.

### Analysis of Syk Protein Levels in Osteoclast Cultures

The different severity of the *in vivo* bone phenotypes (Figures 1–5) and *in vitro* osteoclast developmental defect (Figure 6) between the *Syk*^Δ*OC*^ and *Syk*^Δ*Haemo*^ mutants raised the possibility that Syk is incompletely deleted from *Syk*^Δ*OC*^ osteoclasts. To test this more specifically, we performed Western Blot analysis of Syk expression during osteoclastic and macrophage differentiation of wild type and mutant bone marrow cells.

**Figure 9:**
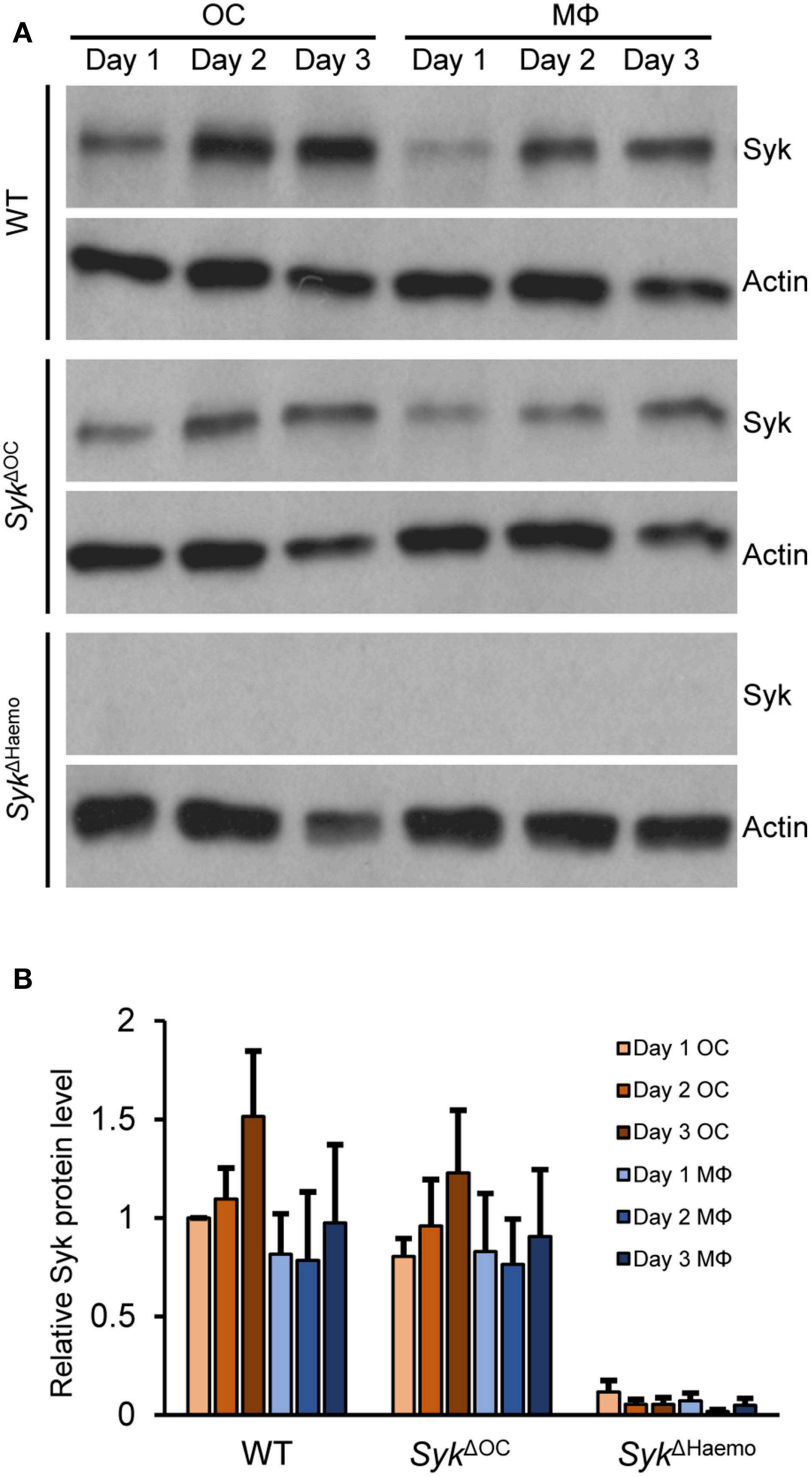
Analysis of the level of Syk protein in osteoclast and macrophage cultures. Bone marrow-derived myeloid progenitors from wild type (WT), *Syk*^Δ*OC*^ or *Syk*^Δ*Haemo*^ mice were cultured in the presence of 50 ng/ml M-CSF with (osteoclasts; OC) or without (macrophages; MΦ) 50 ng/ml RANKL for the indicated times. Whole-cell lysates were then prepared and processed for immunoblotting for Syk, or for actin as a loading control. Representative immunoblots **(A)** or quantification of Syk/actin ratios normalized to Day 1 OC **(B)** are shown. Blots are representative of, and bar graphs show mean and SEM from, 3 to 6 independent experiments.

As shown in Figure 9A, Syk was present in all wild type cultures and its expression slightly even increased during osteoclast differentiation from wild type myeloid progenitors. Importantly, Syk was also present throughout the assessment period in *Syk*^Δ*OC*^ cultures (Figure 9A). On the other hand, Syk was completely absent throughout the entire observation period in *Syk*^Δ*Haemo*^ cultures (Figure 9A). Semiquantitative analysis of the Western blot samples (Figure 9B) confirmed the presence of Syk in all wild type and *Syk*^Δ*OC*^ but not in *Syk*^Δ*Haemo*^ samples. Although there was a tendency of reduced Syk expression in *Syk*^Δ*OC*^ osteoclasts as compared to wild type osteoclasts, this difference was not statistically significant, indicating that the *Syk*^Δ*OC*^ mutation is not able to reduce Syk expression at the overall cell population level.

The above results provided direct evidence supporting our assumption that Syk is incompletely deleted from *Syk*^Δ*OC*^ but it is completely absent from *Syk*^Δ*Haemo*^ osteoclast cultures.

### Genetic Analysis of Syk Deletion During Osteoclastogenesis

One of the possible explanations for the observed differences between the *Syk*^Δ*OC*^ and *Syk*^Δ*Haemo*^ mutants is that Cre expression from the Ctsk-Cre mutation occurs at a late stage of osteoclast development which, combined with the potentially long survival of the Syk protein, leads to reduction of Syk protein levels only at a late stage when osteoclast development has already occurred. The fact that the substantial expression of the *Ctsk* gene (encoding for cathepsin K) begins at 2 days, and is maximal at 3 days after RANKL treatment (Figure 8) (54, 55) would be in line with that possibility.

**Figure 10:**
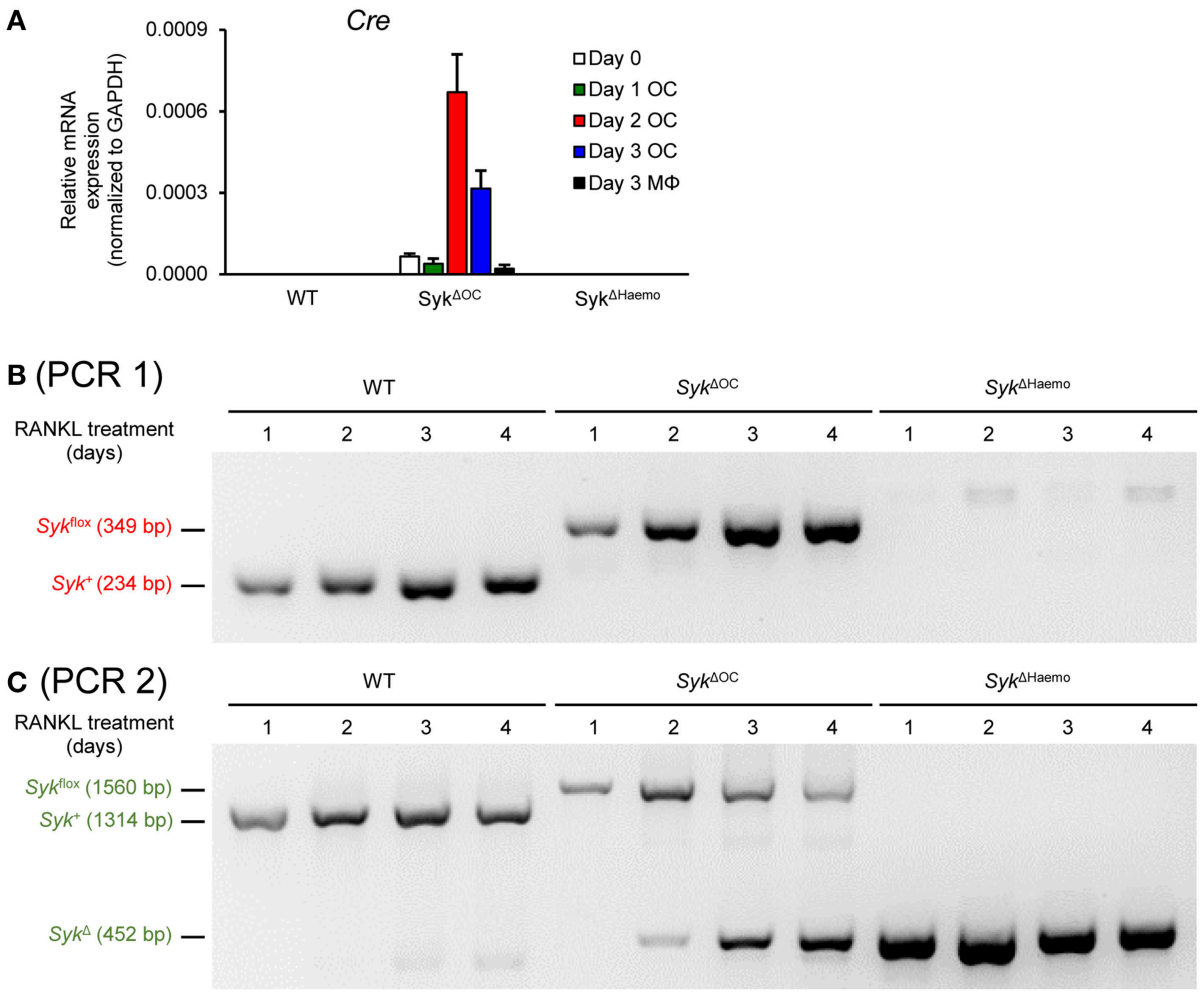
Genetic analysis of Cre expression and Cre-mediated Syk deletion. **(A)** *Cre* expression in wild type (WT), *Syk*^Δ*OC*^ or *Syk*^Δ*Haemo*^ mice bone marrow-derived cells cultured for 0–3 days in the presence of 50 ng/ml M-CSF with (osteoclasts; OC) or without (macrophages; MΦ) 50 ng/ml RANKL. Bar graph shows mean and SEM from 3 independent experiments. **(B,C)** PCR analysis of wild type (WT), *Syk*^Δ*OC*^ or *Syk*^Δ*Haemo*^ osteoclast cultures (differentiated in the presence of 50 ng/ml RANKL and M-CSF for 1–4 days) using PCR 1 (P fwd vs. P rev1 primers; **B**) or PCR 2 (P fwd vs. P rev2 primers; **C**). Images are representative of 4 independent experiments.

As a first approach to address the above issue, we performed qPCR-based analysis of the expression the Cre recombinase in osteoclasts and macrophages from the different genotypes (Figure 10A). As expected, no Cre expression could be observed in wild type cultures. Somewhat surprisingly, no Cre mRNA could be detected in *Syk*^Δ*Haemo*^ cultures either which, together with the complete absence of Syk protein in those cultures (Figure 9) suggests that the Vav-Cre transgene is activated at an early stage of hematopoiesis but it is silenced at the stage of myeloid differentiation tested in our experiments. On the other hand, Cre expression could be readily observed in *Syk*^Δ*OC*^ osteoclast but not macrophage cultures (Figure 10A). Importantly, substantial Cre expression in *Syk*^Δ*OC*^ osteoclasts was first observed 2 days after the initial RANKL treatment, and continued afterwards. Given that a longer time may be needed to the effective deletion of both *Syk* alleles, the supposedly partial deletion efficacy of the Ctsk-Cre transgene and that the Syk mRNA and protein likely does not immediately disappear after the Cre-mediated inactivation of the *Syk* gene, these results are in line with the continued presence of Syk in *Syk*^Δ*OC*^ osteoclasts beyond 2 days after the initial RANKL administration (Figure 9).

As a more direct approach to test Cre-mediated deletion of Syk in our osteoclast cultures, we decided to perform PCR-based analysis of the Syk genomic locus from the cells of our various genotypes. To this end, we first amplified and sequenced the genomic DNA around the two loxP insertion sites, which was used along with the publicly available mouse genomic sequence and the original description of the *Syk*^flox^ mutation (50) to reconstruct the entire sequence of the *Syk*^flox^ allele (Supplementary Figure 1). The organization of the *Syk*^+^ (wild type), *Syk*^flox^ and *Syk*^Δ^ (result of Cre-mediated deletion) alleles is shown in Supplementary Figure 2, indicating the inserted loxP and other sequences, as well as the sites and results of Cre-mediated recombination. Based on this organization, we have designed two PCR protocols (termed PCR 1 and PCR 2) to amplify specific alleles from genomic DNA (Supplementary Figure 2). PCR 1 (Figure 10B and Supplementary Figure 2) was our standard genotyping PCR protocol using the P fwd and P rev1 primer pair, and was able to distinguish between the *Syk*^+^ and the *Syk*^flox^ allele, based on the increased length of the PCR product caused by the 115 bp insertion during the generation of the *Syk*^flox^ allele (49). However, PCR 1 was not able to detect the deleted (*Syk*^Δ^) allele because the sequence corresponding to the P rev1 primer was deleted during Cre-mediated excision of the floxed sequences from the *Syk*^flox^ allele (Supplementary Figure 2). Therefore, we designed a novel PCR protocol (PCR 2; Figure 10C and Supplementary Figure 2) using the same P fwd forward primer along with a new P rev2 reverse primer, spanning the entire floxed sequence, allowing the simultaneous detection of all three (*Syk*^+^, *Syk*^flox^, and *Syk*^Δ^) alleles. We then cultured wild type, *Syk*^Δ*OC*^ and *Syk*^Δ*Haemo*^ bone marrow cells in the presence of M-CSF and RANKL for different periods of time and analyzed their genomic DNA with both the PCR 1 (Figure 10B) and PCR 2 (Figure 10C) protocols.

Results with PCR 1 are shown in Figure 10B. In line with our expectations, the *Syk*^+^ allele was present throughout the assay period in wild type osteoclast cultures and the *Syk*^flox^ allele was present in all *Syk*^Δ*OC*^ samples. Though the latter finding indicated the presence of the non-recombined *Syk*^flox^ allele throughout osteoclast development, it did not exclude substantial deletion (reduction) of the *Syk*^flox^ allele given the tendency of PCR to amplify even small amounts of the target templates when no competing templates are present. In contrast, neither the *Syk*^+^ nor the *Syk*^flox^ allele could be amplified from *Syk*^Δ*Haemo*^ cultures (Figure 10B), suggesting complete deletion of the *Syk*^flox^ allele from those cells, likely in an earlier stage of hematopoietic development. Unfortunately, the *Syk*^Δ^ allele could not be detected with the PCR 1 protocol (Figure 10B and Supplementary Figure 2).

Results with PCR 2 (which could detect all three alleles including the *Syk*^Δ^ allele; see Supplementary Figure 2) is shown in Figure 10C. Those experiments confirmed the expected exclusive presence of the *Syk*^+^ allele throughout the experiment in wild type cultures, as well as the exclusive presence of the *Syk*^Δ^ allele throughout the *Syk*^Δ*Haemo*^ samples, indicating complete deletion of the *Syk*^flox^ allele in the *Syk*^Δ*Haemo*^ cultures. In contrast to the static picture in wild type and *Syk*^Δ*Haemo*^ cultures, the *Syk*^Δ*OC*^ cultures showed dynamic changes in the Syk locus (Figure 10C). While only the *Syk*^flox^ allele was seen 1 day after the initial RANKL treatment, the *Syk*^Δ^ allele appeared and its amount gradually increased during the next 3 days, parallel to a proportional decline (but not complete disappearance) of the *Syk*^flox^ allele (Figure 10C). It should be noted that the appearance of the smaller-size *Syk*^Δ^ allele likely had a competitive advantage over the larger-size *Syk*^flox^ allele in these PCR reactions, leading to a likely underestimation of the amount of the *Syk*^flox^ allele. Taken together, those results and the time course of the changes indicate that Ctsk-Cre-mediated deletion of the *Syk*^flox^ allele occurs gradually during 2–4 days after RANKL addition and that only an incomplete genetic deletion of Syk is achieved even until the end of the observation period.

The above results indicate slow and gradual deletion of the *Syk*^flox^ allele in *Syk*^Δ*OC*^ osteoclast cultures, which is in line with the slow activation of the *Ctsk* gene during *in vitro* osteoclast development (Figures 8, 10A) (54, 55). These results may also explain the less severe *in vivo* phenotypes (Figures 1–5) and less pronounced *in vitro* osteoclast developmental defect (Figure 6), as well as the continuous presence of Syk in osteoclast cultures (Figure 9), in the *Syk*^Δ*OC*^ mutants, as compared with the *Syk*^Δ*Haemo*^ mutants which show early and complete deletion of the *Syk*^flox^ allele from the beginning of the entire osteoclast developmental process.

### Analysis of Myeloid-Specific Syk Deletion

Osteoclasts are derived from early myeloid progenitors through a developmental process related to that of macrophages. Therefore, we have also tested certain aspects of osteoclast biology in *Syk*^Δ*Myelo*^ mutants in which Syk is conditionally deleted using the myeloid-specific LysM-Cre knock-in mutation. The *Syk*^Δ*Myelo*^ mutation strongly reduced (but did not completely abrogate) osteoclast development, both in terms of the number of osteoclasts (Supplementary Figure 3A) and the area covered by osteoclasts (Supplementary Figure 3B). As shown in Supplementary Figure 3C, Syk expression was strongly reduced (but did not completely disappear) in both *Syk*^Δ*Myelo*^ osteoclasts and macrophages. The *Syk*^Δ*Myelo*^ mutation also partially reduced osteoclast-specific gene expression, i.e., the upregulation of the mRNA of the *Tm7sf4, Acp5, Calcr, Nfatc1*, and *Ctsk* genes (Supplementary Figure 3D). We have also tested Cre expression in wild type and *Syk*^Δ*Myelo*^ cells. As shown in Supplementary Figure 3D, Cre mRNA was absent from wild type cells but it was expressed in all *Syk*^Δ*Myelo*^ samples. Interestingly, Cre expression was especially high in early myeloid progenitors (Day 0 samples) and declined afterwards both in osteoclast and macrophage cultures. Taken together, the *Syk*^Δ*Myelo*^ mutation leads to strong but incomplete deletion of Syk during early myeloid differentiation, leading to strongly reduced but not completely abrogated *in vitro* development of osteoclasts.

## Discussion

In this manuscript, we provide direct genetic evidence for the role of the Syk tyrosine kinase in normal bone homeostasis in adult mice. The perinatal lethality of *Syk*^−/−^ mice was overcome by lineage-specific conditional deletion of Syk in osteoclasts (*Syk*^Δ*OC*^ mice) or in the entire hematopoietic system (*Syk*^Δ*Haemo*^ mice). Both osteoclast-specific and hematopoietic Syk deletion led to increased trabecular bone mass and defective *in vitro* osteoclast development and function. However, hematopoietic Syk deletion caused more robust changes than osteoclast-specific Syk deletion both *in vivo* and *in vitro*. Our results suggest that this is due to late and incomplete deletion of Syk in osteoclast-specific Syk mutants, likely caused by late activation and modest activity of Cre expression driven by the *Ctsk* gene promoter during osteoclast development.

We and others have previously shown that Syk plays an important role in *in vitro* osteoclast development and osteoclast-mediated resorptive activity (40, 42, 44). However, the role of Syk in bone homeostasis in live mice could not be tested because of the perinatal lethality of *Syk*^−/−^ mice (17, 18), although bone density appeared to be increased in third-trimester *Syk*^−/−^ fetuses (44). Unfortunately, the *in vitro* osteoclast phenotypes cannot be directly extrapolated to the *in vivo* situation since a number of mutations even within the same pathway, such as DAP12 (38, 41–43) or PLCγ2 (54, 58, 59) deficiency, provide examples of practically complete *in vitro* osteoclast defects despite only moderately increased *in vivo* bone mass. Our *in vivo* results, especially those with the *Syk*^Δ*Haemo*^ mice, provide the first direct genetic evidence for a major and critical role of Syk in bone homeostasis in live animals.

The two main models used in this study clarify different aspects of the role of Syk in bone metabolism: the *Syk*^Δ*OC*^ mice provide evidence for an osteoclast-specific role of Syk but it only leads to limited defects, while the *Syk*^Δ*Haemo*^ mice have the widest Syk deletion without embryonic lethality and therefore show the maximum extent of bone resorption defects.

Despite the clear *in vivo* phenotypes of conditional Syk-deficient mice, a number of questions related to the cell type(s) responsible remain open. Our experiments with the *Syk*^Δ*OC*^ mice indicate that the role of Syk in bone metabolism is at least in part mediated by Syk expression in osteoclasts. However, it is at present unclear why *Syk*^Δ*Haemo*^ mice have a more severe phenotype than the *Syk*^Δ*OC*^ animals. A reasonable explanation, also supported by our *in vitro* findings, is that the *Syk*^Δ*OC*^ mutation only partially deletes Syk in the osteoclast lineage (see further discussion below). However, we cannot exclude the possibility that changes to (a) hematopoietic lineage(s) other than osteoclasts in the *Syk*^Δ*Haemo*^ mice also contribute to the increased bone mass. In addition, it is also possible that Syk deletion in osteoclasts and/or other hematopoietic cells indirectly promote osteoblast-mediated bone production. It should be mentioned that prior studies (44) showed normal bone production by *Syk*^−/−^ osteoblasts, therefore it is unlikely that Syk deficiency in osteoblasts (e.g., through a leaky Cre expression) contributes to the observed *in vivo* bone phenotypes. It should also be noted that our micro-CT studies indicate increased trabecular number rather than a higher trabecular thickness as the main cause of the *in vivo* bone phenotypes. Unfortunately, different groups have reported different contributions of the changes of trabecular number and trabecular thickness to increased bone mass linked to osteoclast defects (42, 43, 54, 55), making it rather difficult to determine the contribution of osteoclasts and osteoblasts to a bone phenotype based on micro-CT data.

An interesting question arising from this study is why the *Syk*^Δ*OC*^ mutation causes a less severe osteoclast phenotype than the *Syk*^Δ*Haemo*^ mutation. Our results clearly indicate that the *Syk*^Δ*OC*^ mutation is less effective in inactivating the Syk gene in osteoclasts. One possible explanation is the fact that the Ctsk-Cre mutation triggers Cre activation at a relatively later time point (starting at ~2 days after RANKL treatment) which, combined with the likely continued presence of the preexisting *Syk* mRNA and Syk protein beyond complete deletion of both Syk alleles, may lead to a late disappearance of the Syk protein at a time point where osteoclast development and osteoclast-mediated bone resorption has already occurred. The activation kinetics of the *Ctsk* gene (Figure 8) and of the Ctsk-Cre mutation (Figure 10A), as well as the late appearance of the *Syk*^Δ^ allele (Figure 10C) all support this explanation. Another possible explanation is that the level of Cre expression from the Ctsk-Cre mutation is too low to provide complete Syk deletion and therefore a significant amount of Syk remains present even after activation of the Ctsk-Cre mutation. In this respect, it is interesting to see that the maximum level of Cre expression in *Syk*^Δ*OC*^ cultures (Figure 10A) is at least an order of magnitude less than that in the *Syk*^Δ*Myelo*^ cultures (Supplementary Figure 3D). Nevertheless, both scenarios and our own results are consistent with prior reports from the literature showing good specificity but incomplete deletion of target genes (incomplete penetrance) by the Ctsk-Cre mutation (54, 55, 60). Those results also point to the fact that the suitability of Cre-expressing mouse strains for the lineage-specific deletion of floxed alleles depends not only on the specificity of the Cre expression but also on its timing, i.e., whether sufficient time is available for nearly complete deletion of the target gene.

Though the main message of our manuscript is the increased *in vivo* bone mass upon conditional deletion of Syk in live mice, some of our results also address the mechanism of the contribution of Syk to osteoclast development and function. While osteoclast-specific gene expression was reduced in *Syk*^Δ*OC*^ and *Syk*^Δ*Myelo*^ cultures, it was not completely abrogated even in *Syk*^Δ*Myelo*^ cells which practically completely lacked Syk protein expression. Therefore, Syk may not only be involved in osteoclast-specific gene expression but maybe also in later processes such as (pre)osteoclast fusion or the osteoclast-mediated resorption process. It is particularly interesting in this respect that DC-STAMP was only moderately affected by Syk deletion, suggesting that a possible role of Syk in (pre)osteoclast fusion may rely on mechanisms other than DC-STAMP expression. It is also worth noting that practically complete defect of matrix resorption was seen in both *Syk*^Δ*OC*^ and *Syk*^Δ*Myelo*^ cultures (i.e., no substantial difference between the two mutations could be seen in this assay), which, however, is complicated by the fact that this assay measures both osteoclast development and the resorptive activity of the cells, and that the longer culture period could have allowed more complete Syk deletion by the Ctsk-Cre mutation. It is also of interest why the number of osteoclasts are reduced on Day 3.5 in the *Syk*^Δ*OC*^ cultures (Figure 6). This may be simply due to the fusion of the cells reducing the number of individual osteoclasts, apoptotic disappearance of osteoclasts during this late stage of culture, and/or active deletion of Syk toward that time period.

We and others have shown that Syk is required for the development of autoantibody-induced arthritis in experimental mice (24, 33–35) and Syk has been proposed as a therapeutic target in human rheumatoid arthritis (61–63). A possible role for Syk in various immune and other cells such as neutrophils, macrophages, mast cells or even platelets (16, 22–24, 26–29, 31, 64–67) may provide an explanation for this observation. Nevertheless, it is important to note that both murine arthritis models (33) and human rheumatoid arthritis (5) are accompanied with bone erosions. Therefore, the role of Syk in osteoclast-mediated *in vivo* bone resorption may also provide an additional cell type beyond immune/inflammatory cells in which Syk inhibitors may have a beneficial therapeutic effect. In addition, Syk-mediated bone resorption may also be a therapeutic target in other diseases characterized by osteoclast-mediated bone resorption such as osteoporosis (4) or osteolytic cancer metastases (7, 8).

Taken together, our results provide direct genetic evidence for the role of Syk in *in vivo* bone metabolism and therefore may contribute to the rationale of developing Syk inhibitors for the treatment of diseases characterized by pathologic bone loss.

## Ethics Statement

All animal experiments were approved by the Animal Experimentation Review Board of the Semmelweis University.

## Author Contributions

DC, DG, and AM conceived the study, designed the experiments, analyzed, and interpreted the data and wrote the manuscript. DC and ES performed most of the experiments. AA and SB performed the qPCR experiments. PA and ZJ performed the histological studies. CD-N conducted the micro-CT scanning. AM supervised the project.

### Conflict of Interest Statement

The authors declare that the research was conducted in the absence of any commercial or financial relationships that could be construed as a potential conflict of interest.
