# Supplementary figures and images for: Hematopoietic or Osteoclast-Specific Deletion of Syk Leads to Increased Bone Mass in Experimental Mice
Source: Front Immunol. 2019 Apr 30;10:937. doi: 10.3389/fimmu.2019.00937 (PMC6524727; doi:10.3389/fimmu.2019.00937)

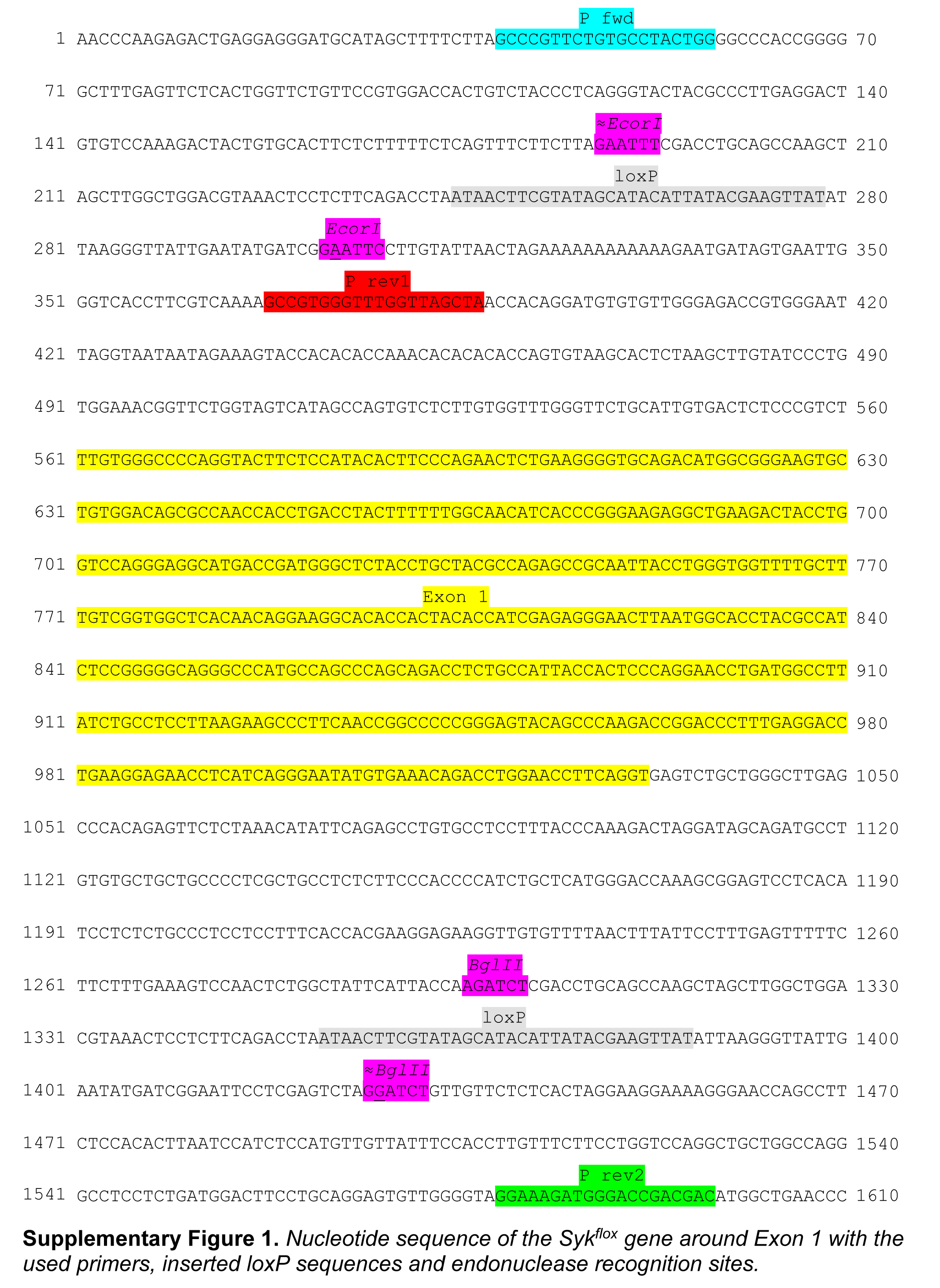

Supplement: Supplementary file 1 [file Image_1.TIF]

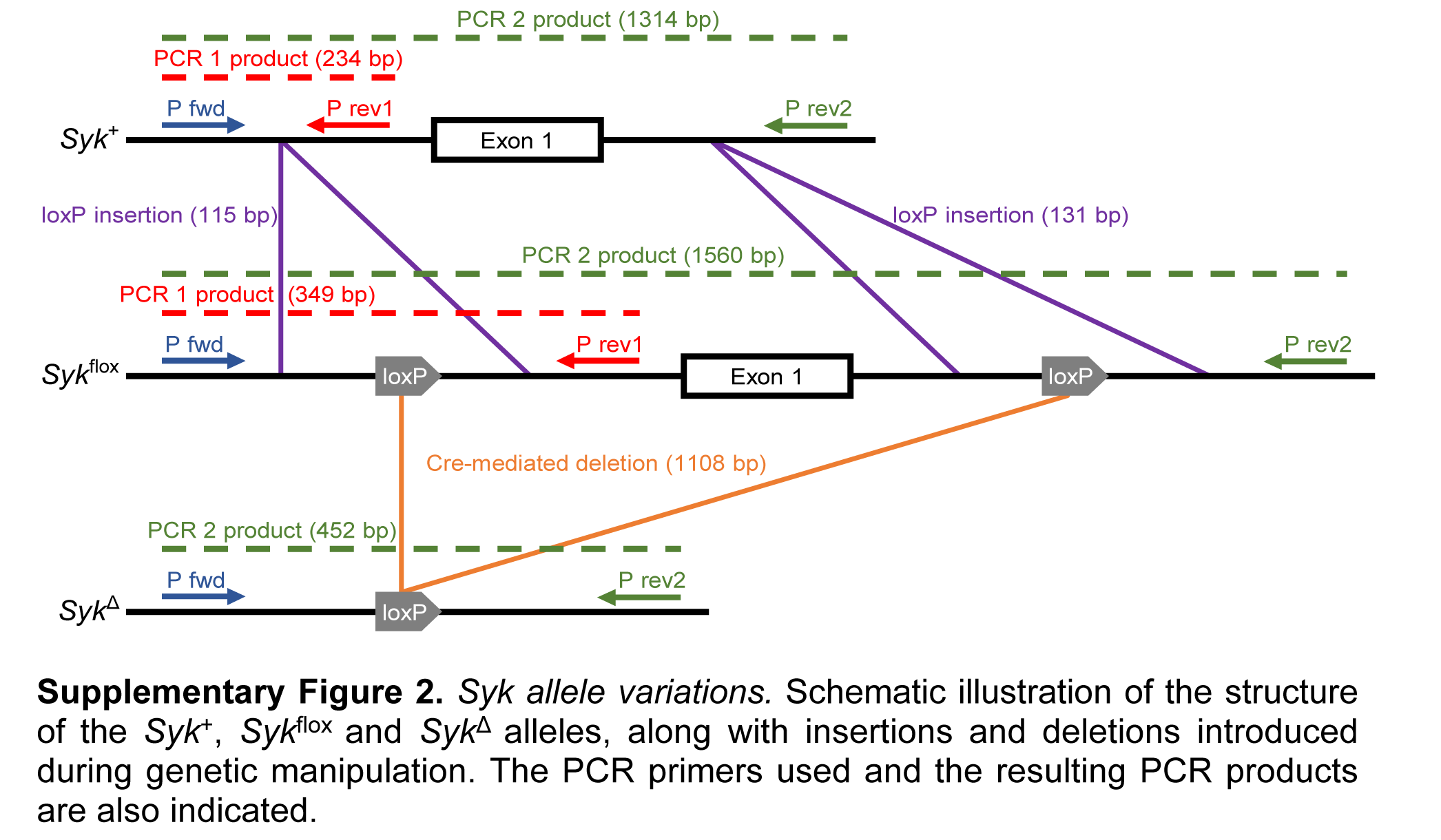

Supplement: Supplementary file 2 [file Image_2.TIF]

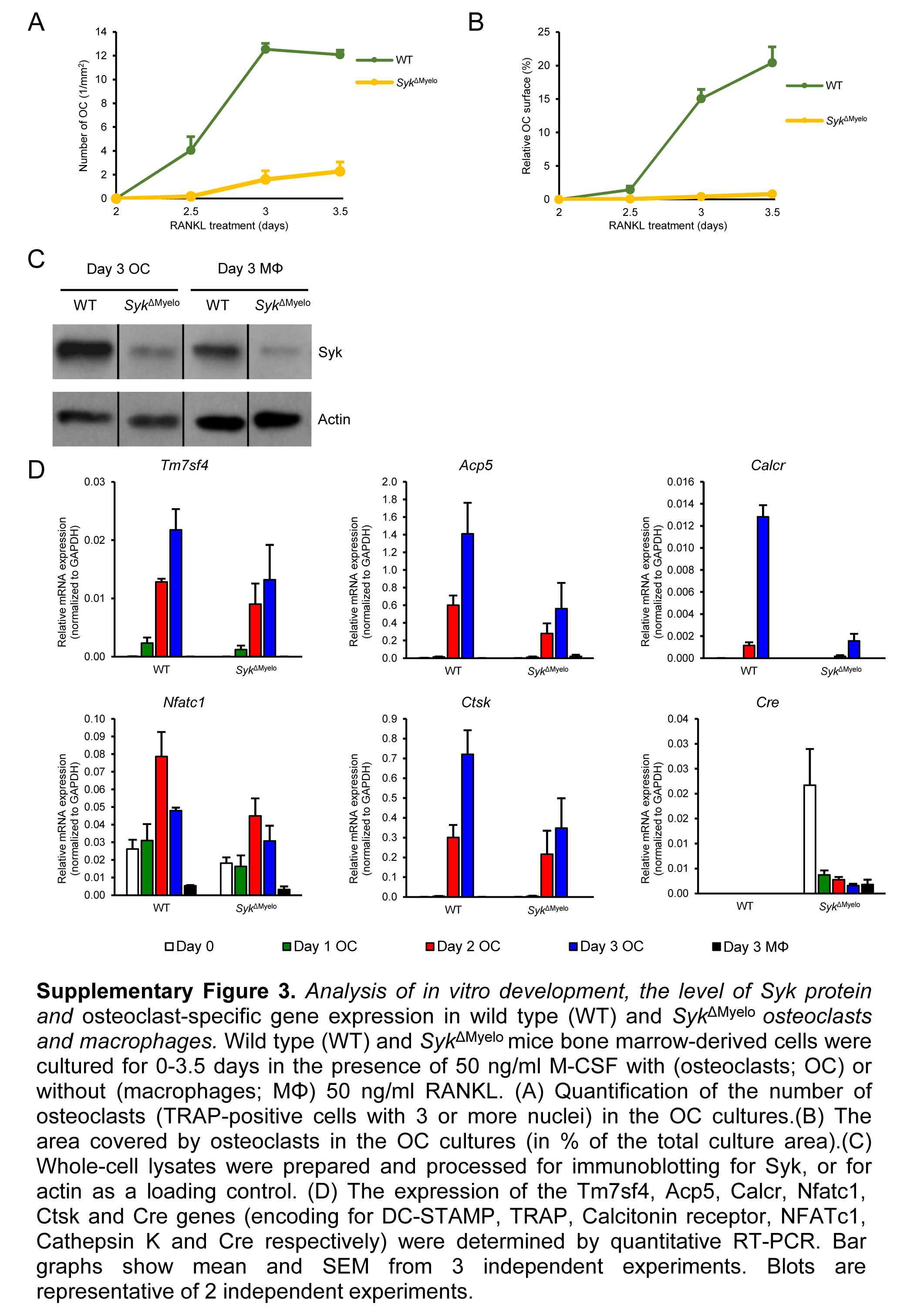

Supplement: Supplementary file 3 [file Image_3.TIF]
